# Supplementary material for: Isoform-specific mutation in Dystonin-b gene causes late-onset protein aggregate myopathy and cardiomyopathy
Source: eLife. 2022 Aug 9;11:e78419. doi: 10.7554/eLife.78419 (PMC9365387; doi:10.7554/eLife.78419)
Supplement: Supplementary file 1. [file elife-78419-supp1.docx]

**Supplementary file 1A** List of up-regulated genes of GO biological process

| GO biological process | -log10 P value | Genes |
| --- | --- | --- |
| cell adhesion | 8.30 | *Cd44*, *Frem2*, *Nuak1*, *Ptk2b*, *Alcam*, *Ackr3*, *Bves*, *Cdh22*, *Comp*, *Col6a2*, *Col7a1*, *Col8a1*, *Col12a1*, *Col16a1*, *Ctgf*, *Cntn2*, *Cntn5*, *Dsc2*, *Dsg2*, *Itga11*, *Itga5*, *Lamb3*, *Ly9*, *Mfap4*, *Mybpc3*, *Mybpc2*, *Postn*, *Pgm5*, *Spp1*, *Sorbs2*, *Svep1*, *Tnc*, *Thbs4*, *Tnfrsf12a*, *Vcan* |
| muscle contraction | 5.82 | *Acta2*, *Lmod2*, *Lmod3*, *Myom1*, *Myom2*, *Mybpc3*, *Mybpc2*, *Myh7*, *Slmap*, *Ttn* |
| response to unfolded protein | 4.77 | *Chac1*, *Ddit3*, *Atf6*, *Hsph1*, *Hspb1*, *Hspa4l*, *Hsp90aa1*, *Mfn2*, *Thbs4* |
| heart development | 4.55 | *Frem2*, *Pdlim3*, *Adam19*, *Alpk3*, *Bves*, *Bmpr1a*, *Cenpf*, *Ednra*, *Fbn1*, *Ift74*, *Mical2*, *Pam*, *Ptn*, *Prkdc*, *Ptpn11*, *Ttn*, *Tgfb2*, *Vcan*, *Xirp2* |
| positive regulation of neuron projection development | 3.54 | *Atp8a2*, *Ndrg4*, *Ptk2b*, *Ankrd1*, *Dpysl3*, *Dbn1*, *Dmd*, *Hspb1*, *Ndnf*, *Ptn*, *Ptprz1*, *Twf2* |
| skeletal muscle tissue development | 3.51 | *Pdlim3*, *Bves*, *Casq1*, *Dmd*, *Myh14*, *Srpk3*, *Svil*, *Vgll2* |
| positive regulation of cell-substrate adhesion | 3.48 | *Col8a1*, *Dbn1*, *Fbln2*, *Itga5*, *Ndnf*, *Ptn*, *Spp1* |
| positive regulation of ERK1 and ERK2 cascade | 3.46 | *Cd44*, *Dnajc27*, *Fbxw7*, *Ndrg4*, *Nox4*, *Ptk2b*, *Ackr3*, *Ccl5*, *Ctgf*, *Ednra*, *Pla2g5*, *Ptpn11*, *Ptpn22*, *Tlr4* |
| cellular response to fibroblast growth factor stimulus | 3.30 | *Cd44*, *Ccl5*, *Col1a1*, *Gclc*, *Myc*, *Postn* |
| sarcomere organization | 3.23 | *Casq1*, *Casq2*, *Lmod2*, *Mybpc3*, *Mylk3*, *Ttn* |
| positive regulation of cardiac muscle contraction | 3.07 | *Ctgf*, *Hsp90aa1*, *Nppa*, *Rgs2* |
| cellular response to mechanical stimulus | 2.72 | *Ankrd1*, *Col1a1*, *Ednra*, *Gclc*, *Nppa*, *Nppb*, *Slc38a2*, *Tlr4* |
| peptidyl-tyrosine dephosphorylation | 2.72 | *Dusp18*, *Ptpn11*, *Ptpn2*, *Ptprz1*, *Ptpro* |
| skeletal muscle contraction | 2.59 | *Hsp90aa1*, *Jsrp1*, *Myh14*, *Myh7*, *Tnnt1* |
| actin filament organization | 2.57 | *Fat1*, *Pdlim3*, *Ptk2b*, *Dbn1*, *Enah*, *Lmod2*, *Lmod3*, *Sorbs2* |
| endodermal cell differentiation | 2.54 | *Col7a1*, *Col8a1*, *Col12a1*, *Itga5*, *Lamb3* |
| striated muscle contraction | 2.47 | *Jsrp1*, *Myh7*, *Ryr3*, *Ttn* |
| positive regulation of apoptotic process | 2.40 | *Adamtsl4*, *Bclaf1*, *Ddit3*, *Nox4*, *Slk*, *Atf6*, *Ankrd1*, *Clu*, *Frzb*, *Pde5a*, *Ptn*, *Prkdc*, *Tlr4*, *Trp53inp1*, *Tgfb2*, *Trim35*, *Tnfrsf12a* |
| cell adhesion mediated by integrin | 2.38 | *Col16a1*, *Fbn1*, *Itga11*, *Itga5* |
| response to hypoxia | 2.37 | *Abat*, *Nox4*, *Ptk2b*, *Ednra*, *Hsp90b1*, *Loxl2*, *Nppa*, *Nppb*, *Pam*, *Postn*, *Tgfb2*, *Ucp2* |
| cardiac muscle contraction | 2.36 | *Actc1*, *Casq2*, *Dmd*, *Mybpc3*, *Myh7*, *Ttn* |
| cardiac muscle tissue morphogenesis | 2.29 | *Actc1*, *Ankrd1*, *Ttn*, *Xirp2* |
| regulation of actin cytoskeleton organization | 2.28 | *Rock1*, *Rock2*, *Baiap2*, *Pam*, *Tgfb2*, *Twf2* |
| regulation of membrane potential | 2.20 | *Bves*, *Cacna1h*, *Dpp6*, *Dmd*, *Grin2c*, *Hcn2*, *Kif5b*, *Popdc3* |
| regulation of heart rate | 2.08 | *Bves*, *Casq2*, *Dmd*, *Mybpc3*, *Myh7* |
| response to heat | 2.05 | *Dnaja4*, *Casq1*, *Gclc*, *Hspa4*, *Hsp90aa1*, *Trp53inp1* |
| intracellular signal transduction | 2.02 | *Akap6*, *Nuak1*, *Arhgef6*, *Rock1*, *Rock2*, *Tiam2*, *Wnk1*, *Adcy5*, *Adcy7*, *Ctgf*, *Hspb1*, *Plcg2*, *Rgs11*, *Rgs6*, *Rp1*, *Stk39*, *Spsb4*, *Twf2* |
| response to wounding | 2.01 | *Grin2c*, *Gpx1*, *Myc*, *Slc1a2*, *Sulf2*, *Tgfb2* |

**Supplementary file 1B** List of up-regulated genes of GO cellular component

| GO cellular component | -log10 P value | Genes |
| --- | --- | --- |
| extracellular matrix | 12.11 | *Adamtsl2*, *Adamtsl4*, *Lmcd1*, *Adamts2*, *Bgn*, *Cilp*, *Comp*, *Clu*, *Col1a1*, *Col6a2*, *Col6a3*, *Col8a1*, *Col12a1*, *Ckap4*, *Fbn1*, *Fbln2*, *Hspb1*, *Hsp90aa1*, *Hsp90b1*, *Hist1h4h*, *Ltbp2*, *Loxl2*, *Mfap4*, *Postn*, *Prkdc*, *Slc25a5*, *Tnc*, *Thbs4*, *Timp1*, *Tgfb2*, *Tubb4b*, *Vcan* |
| Z disc | 10.43 | *Pdlim3*, *Casq2*, *Des*, *Dmd*, *Flnc*, *Hspb1*, *Myo18b*, *Myh7*, *Myot*, *Nexn*, *Palld*, *Pgm5*, *Ryr3*, *Slmap*, *Sorbs2*, *Synpo2*, *Synpo2l*, *Sync*, *Ttn*, *Xirp2* |
| sarcolemma | 9.89 | *Akap6*, *Adcy5*, *Aqp4*, *Bgn*, *Bves*, *Cacna1h*, *Casq1*, *Col6a2*, *Col6a3*, *Des*, *Dmd*, *Flnc*, *Myot*, *Pgm5*, *Kcnd3*, *Ryr3*, *Slmap*, *Slc38a2*, *Sync* |
| proteinaceous extracellular matrix | 9.22 | *Adamtsl2*, *Adamtsl4*, *Adamts2*, *Bgn*, *Cilp*, *Comp*, *Col1a1*, *Col6a2*, *Col6a3*, *Col7a1*, *Col8a1*, *Col12a1*, *Col16a1*, *Ctgf,* *Fbn1*, *Fbln2*, *Lamb3*, *Ltbp2*, *Loxl2*, *Mfap5*, *Mfap4*, *Ndnf*, *Postn*, *Ptn*, *Ptprz1*, *Tnc*, *Thbs4*, *Timp1*, *Vcan* |
| cytoskeleton | 7.62 | *Haus8*, *Clip1*, *Fgd6*, *Ptk2b*, *Arhgap24*, *Rock1*, *Rock2*, *Rpgrip1l*, *Slain2*, *Smek1*, *Acta2*, *Actc1*, *Abra*, *Baiap2*, *Cntrl*, *Ckap4*, *Des*, *Dmd*, *Enah*, *Eif3a*, *Filip1*, *Flnc*, *Hspb1*, *Ivns1abp*, *Igf2bp2*, *Kif5b*, *Lmod2*, *Lrrcc1*, *Mtus2*, *Map4*, *Myom1*, *Myom2*, *Mybpc3*, *Mybpc2*, *Myot*, *Nefm*, *Nexn*, *Palld*, *Pcnt*, *Pgm5*, *Pbxip1*, *Prepl*, *Rp1*, *Rttn*, *Slmap*, *Sept6*, *Stk39*, *Sorbs2*, *Svil*, *Synpo2l*, *Tpr*, *Tppp*, *Tppp3*, *Ttll7*, *Tubb4b*, *Twf2* |
| basement membrane | 5.77 | *Frem2*, *Col4a5*, *Col7a1*, *Col8a1*, *Fbn1*, *Lamb3*, *Loxl2*, *Ptn*, *P3h2*, *Tnc*, *Thbs4*, *Timp1*, *Tgfb2* |
| myofibril | 5.64 | *Abra*, *Ankrd1*, *Ankrd23*, *Casq1*, *Lmod2*, *Mybpc3*, *Myh7*, *Ttn*, *Twf2* |
| filopodium | 5.10 | *Fat1*, *Tiam2*, *Acta2*, *Actc1*, *Baiap2*, *Dbn1*, *Dmd*, *Enah*, *Palld*, *Ptprz1*, *Twf2* |
| perinuclear region of cytoplasm | 4.82 | *Akap6*, *Atp9a*, *Ehd4*, *Fbxw7*, *Fat1*, *Gtpbp4*, *Nox4*, *Ptk2b*, *Rasd1*, *Slk*, *Ackr3*, *Cntrl*, *Cenpf*, *Clu*, *Ctgf*, *Ckap4*, *Dbn1*, *Hsp90aa1*, *Hsp90b1*, *Hhatl*, *Kif5b*, *Myc*, *Nppa*, *Nppb*, *Pam*, *Pla2g5*, *Ptn*, *Ptpn22*, *Ryr3*, *Spp1*, *Sorbs2*, *Sync*, *Tlr4*, *Tppp*, *Twf2* |
| focal adhesion | 4.14 | *Cd44*, *Fat1*, *Nox4*, *Ptk2b*, *Arhgap24*, *Rnd3*, *Actc1*, *Alcam*, *Enah*, *Flnc*, *Hspb1*, *Hsp90b1*, *Itga11*, *Itga5*, *Nexn*, *Palld*, *Pgm5*, *Rhou*, *Rpl3*, *Sorbs2*, *Svil*, *Synpo2*, *Tnc* |
| lamellipodium | 4.09 | *Fat1*, *Ptk2b*, *Arhgef6*, *Rock1*, *Tiam2*, *Acta2*, *Actc1*, *Dpysl3*, *Dbn1*, *Enah*, *Palld*, *Ptprz1*, *Sorbs2*, *Twf2* |
| neuromuscular junction | 4.08 | *Col4a5*, *Des*, *Dok7*, *Nefm*, *Postn*, *Ptn*, *Kcnc3*, *Sync*, *Thbs4* |
| collagen trimer | 4.02 | *Col1a1*, *Col4a5*, *Col6a2*, *Col6a3*, *Col7a1*, *Col8a1*, *Col12a1*, *Col16a1*, *Fam122b*, *Msr1* |
| M band | 4.00 | *Hspb1*, *Lmod2*, *Myom1*, *Myom2*, *Slmap*, *Ttn* |
| I band | 3.80 | *Actc1*, *Ankrd1*, *Ankrd23*, *Casq1*, *Hspb1*, *Ttn* |
| actin cytoskeleton | 3.22 | *Pdlim3*, *Acta2*, *Abra*, *Ankrd23*, *Baiap2*, *Dbn1*, *Enah*, *Flnc*, *Ivns1abp*, *Mylk3*, *Palld*, *Svil*, *Synpo2*, *Synpo2l* |
| cell projection | 3.17 | *Atp8a2*, *Clip1*, *Ptk2b*, *Arhgef6*, *Arhgap24*, *Rock1*, *Rpgrip1l*, *Tiam2*, *Adam22*, *Alcam*, *Adcy5*, *Baiap2*, *Ccdc114*, *Dpysl3*, *Dbn1*, *Enah*, *Enkur*, *Ift74*, *Ngef*, *Palld*, *Palmd*, *Kcnd3*, *Rhou*, *Rp1*, *Rttn*, *Spp1*, *Sorbs2*, *Svil*, *Ttll7*, *Twf2*, *Whrn* |
| axon | 3.17 | *Mycbp2*, *Ptk2b*, *Adam22*, *Alcam*, *Cntn2*, *Hspb1*, *Hcn2*, *Myc*, *Myh14*, *Nefm*, *Palld*, *Pvalb*, *Ptprz1*, *Slc1a2*, *Slc38a2*, *Slc38a7*, *Sptbn4*, *Tgfb2*, *Uchl1*, *Whrn* |
| extracellular exosome | 3.02 | *Abat*, *Nt5e*, *Art3*, *Abcc1*, *Atp6v1h*, *Cd44*, *Ehd4*, *Fat1*, *Frem2*, *Rnd3*, *Sec14l2*, *St3gal6*, *Slk*, *Tiam2*, *Ugp2*, *Acta2*, *Actc1*, *Alcam*, *Bgn*, *Baiap2*, *Cilp*, *Comp*, *Cct3*, *Clu*, *Col6a2*, *Col6a3*, *Col8a1*, *Col12a1*, *Cubn*, *Ckap4*, *Des*, *Dsc2*, *Dsg2*, *Dpp6*, *Eno1*, *Eno1b*, *Fam26e*, *Fbn1*, *Fgl2*, *Fbln2*, *Fstl1*, *Fuca2*, *Gpx1*, *Gyg*, *Golga4*, *Gdf15*, *Hsph1*, *Hspb1*, *Hspa4*, *Hsp90aa1*, *Hsp90b1*, *Hist1h1e*, *Hist1h4h*, *Il18bp*, *Iars*, *Ltbp2*, *Mfap4*, *Map4*, *Mllt3*, *Myh14*, *Pvalb*, *Pi16*, *Pam*, *Plcg2*, *Plekha1*, *Ptpro*, *Rpl3*, *Spp1*, *Shmt1*, *Slc1a4*, *Slc16a1*, *Slc25a5*, *Slc3a1*, *Slc4a4*, *Sptbn4*, *Tom1l1*, *Thbs4*, *Timp1*, *Ttn*, *Tppp3*, *Tubb4b*, *Twf2*, *Uchl1*, *Zfp445* |
| neuronal cell body | 3.01 | *Ptk2b*, *Alcam*, *Bmpr1a*, *Baiap2*, *Cntn2*, *Dpp6*, *Dbn1*, *Hsp90aa1*, *Hcn2*, *Palld*, *Pvalb*, *Pam*, *Pde1c*, *Kcnd3*, *Ptprz1*, *P2rx6*, *Siah2*, *Slc1a4*, *Slc38a2*, *Slc38a7*, *Sorbs2*, *Sptbn4*, *Tgfb2*, *Uchl1*, *Whrn* |
| neuron projection | 2.89 | *Abat*, *Pacrg*, *Pdzd2*, *Baiap2*, *Clu*, *Cntn2*, *Eno1*, *Eno1b*, *Fam126a*, *Gnao1*, *Hsp90aa1*, *Kif5b*, *Map4*, *Nefm*, *Pam*, *Kcnd3*, *Rgs2*, *Siah2*, *Slc1a2*, *Sstr3*, *Sv2b* |
| myelin sheath | 2.89 | *Cct3*, *Cntn2*, *Eno1*, *Eno1b*, *Gnao1*, *Hsp90aa1*, *Myh14*, *Nefm*, *Slc25a5*, *Syn2*, *Tppp*, *Tubb4b*, *Uchl1* |
| sarcomere | 2.89 | *Actc1*, *Abra*, *Lmod2*, *Myom1*, *Mybpc3*, *Ttn* |
| sarcoplasmic reticulum | 2.74 | *Akap6*, *Rasd1*, *Casq1*, *Casq2*, *Jsrp1*, *Ryr3*, *Thbs4* |
| extracellular region | 2.62 | *Abat*, *Nt5e*, *Art3*, *Abcc1*, *Atp6v1h*, *Cd44*, *Ehd4*, *Fat1*, *Frem2*, *Rnd3*, *Sec14l2*, *St3gal6*, *Slk*, *Tiam2*, *Ugp2*, *Acta2*, *Actc1*, *Alcam*, *Bgn*, *Baiap2*, *Cilp*, *Comp*, *Cct3*, *Clu*, *Col6a2*, *Col6a3*, *Col8a1*, *Col12a1*, *Cubn*, *Ckap4*, *Des*, *Dsc2*, *Dsg2*, *Dpp6*, *Eno1*, *Eno1b*, *Fam26e*, *Fbn1*, *Fgl2*, *Fbln2*, *Fstl1*, *Fuca2*, *Gpx1*, *Gyg*, *Golga4*, *Gdf15*, *Hsph1*, *Hspb1*, *Hspa4*, *Hsp90aa1*, *Hsp90b1*, *Hist1h1e*, *Hist1h4h*, *Il18bp*, *Iars*, *Ltbp2*, *Mfap4*, *Map4*, *Mllt3*, *Myh14*, *Pvalb*, *Pi16*, *Pam*, *Plcg2*, *Plekha1*, *Ptpro*, *Rpl3*, *Spp1*, *Shmt1*, *Slc1a4*, *Slc16a1*, *Slc25a5*, *Slc3a1*, *Slc4a4*, *Sptbn4*, *Tom1l1*, *Thbs4*, *Timp1*, *Ttn*, *Tppp3*, *Tubb4b*, *Twf2*, *Uchl1*, *Zfp445* |
| myosin filament | 2.57 | *Myom1*, *Mybpc3*, *Mybpc2*, *Myh7* |
| growth cone | 2.52 | *Ptk2b*, *Tiam2*, *Clu*, *Dpysl3*, *Dbn1*, *Myh14*, *Ngef*, *Palld*, *Ptprz1*, *Twf2*, *Whrn* |
| cell junction | 2.51 | *Fat1*, *Gid8*, *Nox4*, *Ptk2b*, *Arhgap24*, *Rpgrip1l*, *Bves*, *Dsc2*, *Dsg2*, *Dok7*, *Dbn1*, *Dmd*, *Enah*, *Grin2c*, *Itga5*, *Nexn*, *Palld*, *Pgm5*, *Pja2*, *Ptpn11*, *P2rx6*, *Rhou*, *Scamp5*, *Sorbs2*, *Svil*, *Syn2*, *Sv2b*, *Syt12*, *Xirp2* |
| stress fiber | 2.43 | *Nox4*, *Enah*, *Myh14*, *Myh7*, *Palld*, *Pgm5*, *Ptpn11* |
| cell surface | 2.41 | *Nt5e*, *Cd44*, *Ackr3*, *Bgn*, *Clu*, *Cntn2*, *Corin*, *Ckap4*, *Dsg2*, *Dpp6*, *Dmd*, *Enpp1*, *Hsp90aa1*, *Itga5*, *Ly9*, *Pam*, *Pla2g5*, *Ptn*, *Ptgfrn*, *Slc1a2*, *Slc1a4*, *Sulf2*, *Tlr4*, *Tgfb2*, *Tnfrsf12a*, *Vcan* |
| cilium | 2.28 | *Pacrg*, *Rpgrip1l*, *Adcy5*, *Ccdc114*, *Enkur*, *Hk1*, *Ift74*, *Pde1c*, *Rp1*, *Rttn*, *Sstr3*, *Spa17*,  *Ttll7*, *Whrn* |
| intercalated disc | 2.27 | *Akap6*, *Ankrd23*, *Des*, *Dsc2*, *Dsg2*, *Pgm5* |
| postsynaptic density | 2.12 | *Cap2*, *Pdzd2*, *Ptk2b*, *Baiap2*, *Dbn1*, *Grin2c*, *Map4*, *Nefm*, *Pja2*, *P2rx6*, *Sorbs2*, *Syn2*, *Snap91* |
| cell body | 2.11 | Pacrg, Ptk2b, Acta2, Actc1, Cct3, Dpysl3, Gnao1, Hspa1l |
| cytosol | 2.06 | *Chac1*, *Dnaja4*, *Ndrg4*, *Pacrg*, *Sec14l2*, *Wnk1*, *Ankrd1*, *Afmid*, *Atg13*, *Baiap2*, *Cast*, *Car3*, *Clu*, *Ctgf*, *Cdo1*, *Cox19*, *Dpysl3*, *Ppip5k2*, *Enah*, *Fam126a*, *Fam126b*, *Gclc*, *Gpx1*, *Hsph1*, *Hspa1l*, *Hspa4l*, *Hspa4*, *Hsp90aa1*, *Hsp90b1*, *Hk1*, *Irs1*, *Iars*, *Kif5b*, *Lars*, *Msr1*, *Mfn2*, *Mylk3*, *Ngef*, *Ncf4*, *Pam*, *Pde5a*, *Plcg2*, *Piwil2*, *Prepl*, *Prkag3*, *Ppp2r2c*, *Ptpn11*, *Rgs2*, *Rgs6*, *Shmt1*, *Stk39*, *Siah2*, *Sync*, *Tom1l1*, *Trp53inp1*, *Uchl1* |

**Supplementary file 1C** List of up-regulated genes of KEGG pathway

| KEGG pathway | -log10 P value | Genes |
| --- | --- | --- |
| ECM-receptor interaction | 6.39 | *Cd44*, *Comp*, *Col1a1*, *Col4a5*, *Col6a2*, *Col6a3*, *Itga11*, *Itga5*, *Lamb3*, *Spp1*, *Sv2b*, *Tnc*, *Thbs4* |
| Dilated cardiomyopathy | 4.92 | *Actc1*, *Adcy5*, *Adcy7*, *Cacna2d3*, *Des*, *Dmd*, *Itga11*, *Itga5*, *Mybpc3*, *Ttn*, *Tgfb2* |
| Hypertrophic cardiomyopathy (HCM) | 4.28 | *Actc1*, *Cacna2d3*, *Des*, *Dmd*, *Itga11*, *Itga5*, *Mybpc3*, *Prkag3*, *Ttn*, *Tgfb2* |
| Focal adhesion | 3.77 | *Rock1*, *Rock2*, *Comp*, *Col1a1*, *Col4a5*, *Col6a2*, *Col6a3*, *Flnc*, *Itga11*, *Itga5*, *Lamb3*, *Mylk3*, *Spp1*, *Tnc*, *Thbs4* |
| Calcium signaling pathway | 3.24 | *Ptk2b*, *Adcy7*, *Cacna1h*, *Ednra*, *Grin2c*, *Mylk3*, *Pde1c*, *Plcg2*, *Phkg1*, *Ptgfr*, *P2rx6*, *Ryr3*, *Slc25a5* |
| PI3K-Akt signaling pathway | 2.74 | *Comp*, *Col1a1*, *Col4a5*, *Col6a2*, *Col6a3*, *Hsp90aa1*, *Hsp90b1*, *Irs1*, *Itga11*, *Itga5*, *Lamb3*, *Myc*, *Ppp2r3a*, *Ppp2r2c*, *Spp1*, *Tnc*, *Thbs4*, *Tlr4* |
| cGMP-PKG signaling pathway | 2.51 | *Rock1*, *Rock2*, *Adcy5*, *Adcy7*, *Ednra*, *Irs1*, *Mylk3*, *Nppb*, *Pde5a*, *Rgs2*, *Slc25a5* |
| Protein digestion and absorption | 2.51 | *Col1a1*, *Col4a5*, *Col6a2*, *Col6a3*, *Col7a1*, *Col12a1*, *Slc3a1*, *Slc38a2* |
| Arrhythmogenic right ventricular cardiomyopathy (ARVC) | 2.47 | *Cacna2d3*, *Des*, *Dsc2*, *Dsg2*, *Dmd*, *Itga11*, *Itga5* |
| Oxytocin signaling pathway | 2.24 | *Rock1*, *Rock2*, *Adcy5*, *Adcy7*, *Cacna2d3*, *Gnao1*, *Mylk3*, *Prkag3*, *Rgs2*, *Ryr3* |
| Vascular smooth muscle contraction | 2.17 | *Rock1*, *Rock2*, *Acta2*, *Adcy5*, *Adcy7*, *Ednra*, *Mylk3*, *Pla2g2c*, *Pla2g5* |

**Supplementary file 1D** List of down-regulated genes of GO biological process

| GO biological process | -log10 P value | Genes |
| --- | --- | --- |
| cell adhesion | 4.92 | *Egfl7*, *Ephb1*, *Amtn*, *Ctnnal1*, *Col6a6*, *Fblim1*, *Jam3*, *Klra9*, *Lama5*, *Muc4*, *Nid2*, *Prkd2*, *Pcdh17*, *Ret*, *Stab2*, *Thbs2*, *Tmem8*, *Vtn* |
| metabolic process | 4.07 | *Bdh1*, *Acaa2*, *Acsf2*, *Aldh1l1*, *Alad*, *Amy1*, *Bckdha*, *Echdc3*, *Ech1*, *Eci1*, *Fah*, *Gsta2*, *Gstp2*, *Hadhb*, *Isoc2a*, *Man2a2*, *Pah* |
| fatty acid metabolic process | 2.68 | *H2-Ke6*, *Acaa2*, *Acsf2*, *Cyb5a*, *Ech1*, *Eci1*, *Hadhb*, *Ptgds* |
| regulation of ion transmembrane transport | 2.35 | *Kcnip2*, *Cacna1s*, *Cacng6*, *Kcnj8*, *Scn4b*, *Scn4a*, *Stom* |
| muscle organ development | 2.24 | *Dtnbp1*, *Id3*, *Ky*, *Lama5*, *Mtss1* |
| angiogenesis | 2.20 | *Egfl7*, *Ephb1*, *Egf*, *Enpep*, *Jam3*, *Plxnd1*, *Prkd2*, *Tie1*, *Vash1* |
| positive regulation of endothelial cell proliferation | 2.14 | *Egfl7*, *Nrarp*, *Cav2*, *Ccl11*, *Prkd2* |

**Supplementary file 1E** List of down-regulated genes of GO cellular component

| GO cellular component | -log10 P value | Genes |
| --- | --- | --- |
| extracellular exosome | 5.72 | *Abcb1a*, *C1qtnf9*, *Ctdsp1*, *Ephb1*, *Nqo1*, *Sh3gl2*, *Steap4*, *Abhd8*, *Acaa2*, *Ak4*, *Aldh1l1*, *Aldob*, *Alad*, *Amy1*, *Ano1*, *Creg1*, *Cyb5a*, *Eno3*, *Ech1*, *Eci1*, *Egf*, *Efemp1*, *Epn3*, *Fscn1*, *Fabp3*, *Fah*, *Gfra1*, *Gpt*, *Enpep*, *Gsta2*, *Gstp2*, *Gnb3*, *Hpn*, *Hadhb*, *Lama5*, *Mme*, *Mpst*, *Mapk3*, *Mylk4*, *Nid2*, *Nudt14*, *Pxdn*, *Pah*, *Prps2*, *Pdgfrb*, *Prom2*, *Ptgds*, *Ppm1l*, *Rtn4r*, *Rarres2*, *Rps16*, *Scarb1*, *Slc25a25*, *Stom*, *Stxbp1*, *Tmem256*, *Tmem8*, *Tnfsf10*, *Vtn* |
| integral component of plasma membrane | 3.82 | *Cd200*, *Ephb1*, *Rhd*, *Steap4*, *Adra1b*, *Cav2*, *Hpn*, *Il17re*, *Jag2*, *Notch3*, *Plxnb1*, *Plxnd1*, *Kcnj8*, *Prom2*, *Ret*, *Rtn4r*, *Sgca*, *Scarb1*, *Slc22a3*, *Slc26a10*, *Slc38a3*, *Slc6a8*, *S1pr3*, *Stab2*, *Stom*, *Tspan13*, *Tmem150c*, *Tmem8*, *Tnfrsf21* |
| mitochondrion | 2.19 | *Bdh1*, *Atp5g1*, *Abcc9*, *H2-Ke6*, *Macrod1*, *C030006K11Rik*, *Acaa2*, *Acsf2*, *Ak4*, *Aldh1l1*, *Bckdha*, *Cyb5a*, *Cox4i2*, *Cox6b2*, *Echdc3*, *Ech1*, *Eci1*, *Gstp2*, *Hadhb*, *Isoc2a*, *Mpst*, *Mrpl14*, *Mrps28*, *Mapk3*, *Pxmp2*, *Kcnj8*, *Pdp2*, *Sars2*, *Slc25a25*, *Stom*, *Stxbp1*, *Uqcrq*, *Urah* |

**Supplementary file 1F** List of down-regulated genes of KEGG pathway

| KEGG pathway | -log10 P value | Genes |
| --- | --- | --- |
| Metabolic pathways | 2.85 | *Bdh1*, *Atp5g1*, *Cds1*, *H2-Ke6*, *Ndufa4l2*, *Acaa2*, *Adi1*, *Ak4*, *Aox3*, *Aldob*, *Alad*, *Amy1*, *Bckdha*, *Ckm*, *Cox4i2*, *Cox6b2*, *Eno3*, *Fah*, *Ggt5*, *Gpt*, *Hadhb*, *Impa2*, *Man2a2*, *Mpst*, *Nos2*, *Pah*, *Prps2*, *Ptgds*, *Uqcrq*, *Urah* |
| Alzheimer’s disease | 2.66 | *Atp5g1*, *Ndufa4l2*, *Apbb1*, *Cacna1s*, *Cox4i2*, *Cox6b2*, *Mme*, *Mapk3*, *Uqcrq* |
| Cardiac muscle contraction | 2.48 | *Cacna1s*, *Cacng6*, *Cox4i2*, *Cox6b2*, *Myl3*, *Uqcrq* |
| Focal adhesion | 2.24 | *Cav2*, *Col6a6*, *Egf*, *Lama5*, *Mapk3*, *Mylk4*, *Pdgfrb*, *Thbs2*, *Vtn* |

**Supplementary file 1G** Variants of *DST* gene identified in Japanese patients with myopathy

|  | Age | Sex0 | Pathological interpretation | ExonicFunc.ensGene | Variant |
| --- | --- | --- | --- | --- | --- |
| Patient 1 | 11m | F | Congenital neuromuscular disease with uniform type 1 fiber | nonsynonymous SNP | exon40:c.8806C>T:p.P2936S |
|  |  |  |  | nonsynonymous SNP | exon52:c.13390A>C :p.K4464Q |
| Patient 2 | 49y | M | Amyloid myopathy | synonymous SNP | exon40:c.6666C>T:p.Y2222= |
|  |  |  |  | nonsynonymous SNP | exon74:c.18901A>G:p.N6301D |
